# Intimate partner violence against married and cohabiting women in sub-Saharan Africa: does sexual autonomy matter?

**DOI:** 10.1186/s12978-022-01382-1

**Published:** 2022-03-28

**Authors:** Richard Gyan Aboagye, Louis Kobina Dadzie, Francis Arthur-Holmes, Joshua Okyere, Ebenezer Agbaglo, Bright Opoku Ahinkorah, Abdul-Aziz Seidu

**Affiliations:** 1 Department of Family and Community Health, School of Public Health, University of Health and Allied Sciences, Hohoe, Ghana; 2 Department of Population and Health, University of Cape Coast, Cape Coast, Ghana; 3 Department of Sociology and Social Policy, Lingnan University, 8 Castle Peak Road, Tuen Mun, Hong Kong; 4 Department of English, University of Cape Coast, Cape Coast, Ghana; 5 School of Public Health, Faculty of Health, University of Technology Sydney, Sydney, Australia; 6 Centre for Gender and Advocacy, Takoradi Technical University, Takoradi, Ghana; 7 College of Public Health, Medical and Veterinary Sciences, James Cook University, Townsville, Australia

**Keywords:** Intimate partner violence, Sexual autonomy, Sub-Saharan Africa, Demographic and Health Survey

## Abstract

**Background:**

Literature shows that women’s sexual autonomy, which refers to women’s capacity to refuse sex and ask a partner to use condom, has significant implications on the sexual and reproductive health outcomes and sexual-and-gender based violence. Nevertheless, there is scarcity of empirical evidence to support the association between women’s sexual autonomy and intimate partner violence (IPV) in sub-Saharan Africa.

**Methods:**

Data for the study were extracted from the recent Demographic and Health Surveys in 24 countries in sub-Saharan Africa between 2010 and 2019. Bivariable and multivariable binary logistic regression analyses were performed to examine the association between sexual autonomy and IPV in all the studied countries. Statistical significance was set at p < 0.05.

**Results:**

The pooled prevalence of IPV and sexual autonomy in the 24 countries were 38.5% and 73.0% respectively. Overall, the odds of exposure to IPV were higher among women with sexual autonomy, compared to those without sexual autonomy even after controlling for covariates (age, level of education, marital status, current working status, place of residence, wealth quintile and media exposure). At the country-level, women from Angola, Cameroon, Chad, Gabon, Cote d’lvoire, Gambia, Mali, Nigeria, Kenya, Comoros, Zambia, and South Africa who had sexual autonomy were more likely to experience IPV whilst those in Burundi were less likely to experience IPV. The study showed that sexual autonomy increases women’s exposure to IPV and this occurred in many countries except Burundi where women with sexual autonomy were less likely to experience IPV.

**Conclusion:**

The findings highlight the need for serious programs and policies to fight against IPV in the sub-region. Additionally, laws need to be passed and implemented, with law enforcement agencies provided with the necessary resources to reduce intimate partner violence among women with sexual autonomy.

**Supplementary Information:**

The online version contains supplementary material available at 10.1186/s12978-022-01382-1.

## Plain language summary

Globally, intimate partner violence is regarded as a public health concern due to its devastating effects on the physical, emotional, and reproductive health of women. This study sought to determine how women’s capacity to refuse sex, negotiate for safe sex practices such as insisting on partner to use condom, and feeling justified in asking a partner to use condom is associated with the potential of experiencing violence from an intimate partner. Using data from the demographic and health survey conducted between 2010 and 2019, we found that women who had more capacity to refuse sex, negotiate for safe sex practices such as insisting on partner to use condom, and feeling justified in asking a partner to use condom were more likely to experience violence from their intimate partners after controlling for other factors such as the age of the woman, level of education, marital status, place of residence, economic status, and media exposure. The results highlight the need for sub-Saharan African countries to step up programs that ease up intimate partner violence reporting and access to legal support for those who experience it. Additionally, laws need to be passed and implemented, with law enforcement agencies provided with the necessary resources to reduce intimate partner violence among women with sexual autonomy.

**Supplementary Information:**

The online version contains supplementary material available at 10.1186/s12978-022-01382-1.

## Introduction

Intimate partner violence (IPV) is a public health concern and increasingly gaining global attention [1]. Any violence (physical, psychological, and sexual harm) that occurs in an intimate relationship can be considered or referred to as IPV [2–4]. However, in this study, IPV is conceptualized as physical violence (i.e., throwing something at a woman, slapping, punching, threatening with a weapon, kicking or dragging, strangling and pulling of hair), emotional violence (i.e., insults, humiliation and threats to harm) and sexual violence (physically forced into unwanted sex, being forced into other unwanted sexual acts, and physically forced to perform sexual acts that one doesn’t want to). The pervasiveness of IPV worldwide cannot be downplayed as one out of every three females would have ever experienced some form of IPV at some point in their lives [2]. The phenomenon is endemic in sub-Saharan Africa (SSA), with most women being at risk of experiencing IPV [5].

There is the need for more pragmatic and evidence-based approaches towards combating IPV in SSA due to the concomitant harmful consequences of IPV on the health and psychosocial wellbeing of women. IPV infringes on the rights of women and subsequently lowers their confidence and self-efficacy [6, 7]. Moreover, IPV is associated with many negative mental health outcomes including depression, heightened anxiety, and post-traumatic stress [8]. Women who experience IPV are most likely to have poor maternal and child health outcomes as they would not be able to seek early health care due to fear of being abused by their partners [9].  Considering the critical nature of IPV, the United Nations (UN) in its 17 Sustainable Development Goals [SDGs] re-emphasized the need to reduce and possibly eliminate IPV by 2030.

Also, given the relevance and timeliness of the need for evidence-based approach towards the fight against IPV, different researchers have investigated widely this phenomenon to aid policy and intervention development, and implementation [6–8, 10–14]. Most of these studies have investigated how socio-demographic characteristics such age, marital status, educational attainment, and employment status of women predict the likelihood of experiencing IPV as a woman [8, 15, 16]. However, in the context of SSA, studies investigating the influence of other critical factors, such as autonomy on the women’s probability of experiencing IPV have been sparse.

Sexual autonomy describes the independent decision-making related to issues of sex and its related activities. Our study’s conceptualization of women’s sexual autonomy is premised on Budu et al. [17] definition which holds that women’s sexual autonomy refers to a woman’s capacity to refuse sex, negotiate for safe sex practices such as insisting on partner to use condom, and feeling justified in asking a partner to use condom. Literature is replete with evidence that women with high sexual autonomy have significantly lower risk of adverse SRH outcomes including unwanted pregnancy [18] and non-use of modern contraceptives [19]. Amidst the preponderance of evidence establishing association between sexual autonomy and adverse SRH outcomes, there is paucity of information about the association between sexual autonomy and women’s likelihood of experiencing IPV. However, a study conducted in Nigeria [20] reported that women's sexual autonomy, which encompasses women’s ability to ask for condom during sex and refuse sex, has been associated with  higher likelihood of women experiencing IPV. However, the findings of the study could not be generalized to the entire sub-Saharan African countries given the contextual differences. We, therefore, sought to fill the gap in literature and contribute to the use of empirical evidence in the fight against IPV by examining the association between sexual autonomy and IPV against married and cohabiting women in SSA. We hypothesize that women’s sexual autonomy is inversely associated with exposure to  IPV. Findings from our study will inform policies and  interventions aimed at eliminating IPV among married and cohabiting women through women empowerment initiatives. 

## Methods

### Data source and study design

This study pooled data from the demographic and health survey (DHS) of 24 countries in SSA, which adopted a cross-sectional study design. DHS is a nationally representative survey that collects data on several health indicators including IPV and sexual autonomy across low- and middle-income countries. DHSs are mostly carried out every five years. However, the period can be extended in some countries due to certain conditions that exist in such countries. Data for each survey is collected from both men and women, which are sampled by a two-stage sampling technique [21]. The first stage involves the selection of clusters usually called enumeration areas (EAs). The second stage includes choosing of households for the survey. Sampling methodology and data collection procedure used by the DHS are found in a previous study [21]. Data collection was done by survey staff who were trained and instructed in standard DHS procedures. These procedures include general interviewing techniques, conducting interviews at the household level, measuring blood pressures and review of each question and mock interviews between participants. The DHS in SSA is usually conducted in English, French and Portuguese, depending on the official language of the country. For this study, the inclusion criteria were countries whose datasets were published between 2010 and 2019 and had information on the DHS domestic violence modules and sexual autonomy. Using the inclusion criteria, the DHS of 26 countries were initially identified. However, only the DHS of 24 countries had data on IPV and sexual autonomy. The two countries excluded in the study were Benin and Tanzania. Based on eligible respondents for the domestic violence modules [22], only ever married, currently married and cohabiting women were included. In all, 99,769 ever married, currently married and cohabiting women who completed information on IPV and sexual autonomy were included in this study. Table 1 shows the countries that were included in this study. The dataset is freely accessible via this link: https://dhsprogram.com/data/available-datasets.cfm. This paper was prepared in line with Strengthening Reporting of Observational studies in Epidemiology (STROBE) reporting guidelines (Additional file 1: Table S1) [23].

**Table 1:** Description of the sample

Countries	Year of survey	Weighted N	Weighted %
Central Africa			
Angola	2015–16	7466	7.5
Cameroon	2018	3690	3.7
Chad	2014–15	2449	2.4
Congo DR	2013–14	4427	4.4
Gabon	2012	3003	3.0
West Africa			
Burkina Faso	2010	9658	9.7
Cote d’lvoire	2011–12	4170	4.2
Gambia	2013	3139	3.1
Mali	2018	3213	3.2
Nigeria	2018	8161	8.2
Sierra Leone	2019	3477	3.5
Togo	2013–14	4471	4.5
East Africa			
Burundi	2016–17	5897	5.9
Comoros	2012	1745	1.7
Ethiopia	2016	4030	4.0
Kenya	2014	3468	3.5
Mozambique	2011	1964	2.0
Rwanda	2014–15	1479	1.5
Uganda	2016	5964	6.0
Southern Africa			
Malawi	2015–16	4453	4.5
Namibia	2013	875	0.9
South Africa	2016	1880	1.9
Zambia	2018	5788	5.8
Zimbabwe	2015	4902	4.9
All countries		99,769	100.0

### Study variables

#### Outcome variable

IPV was the dependent variable in this study. It was derived from three key variables namely, physical, emotional and sexual violence. These variables focused on a number of questions in the domestic violence module (DVM). However, questions in the DVM were derived from a modified version of the conflict tactics scale [24, 25]. On physical violence (PV), each respondent was asked whether her last partner ever pushed her; shook or threw something at her; slapped her; punched her with his fist or something harmful; kicked or dragged her; strangled or burnt her; threatened her with a knife, gun, or other weapons; and twisted her arm or pulled her hair. For emotional violence (EV), respondents were asked whether their last partner ever: humiliated her; threatened to harm her; and insulted or made her feel bad. Lastly, on sexual violence (SV), respondents were asked whether their partner ever physically forced the respondent into unwanted sex; whether the partner ever forced her into other unwanted sexual acts; and whether the respondent has been physically forced to perform sexual acts which she did not want to. For these questions, the responses were ‘yes’ and ‘no’. A respondent who had experienced at least one of the violent acts was considered as ever experienced physical, emotional or sexual violence. Ever had IPV was generated from all the questions asked on physical, emotional and sexual violence, and respondents who had encountered experienced at least one of these violent acts regarded as ever had IPV and otherwise. Similar coding has been used in previous studies that used DHS dataset [9, 26].

#### Key explanatory variable

The main explanatory variable for this study was sexual autonomy. This variable was created as an index of two questions consisting of “whether married/cohabiting women can refuse sex” and “whether married/cohabiting women can ask their partners to use condoms.” 1 = No; 2 = Yes; and 3 = don't know/not sure/depends were the answer choices in both questions. For this study, the respondents who responded “Don't know/not sure/depends” were dropped. Therefore, the final response options used in the analysis were 1 = No; and 2 = Yes. To create the variable “sexual autonomy” respondents who answered “Yes” to at least one of the questions was considered as having sexual autonomy while those who answered “No” to the two questions were considered as not having sexual autonomy. The selection of the variables and their recoding were informed by literature and availability in the dataset [27–29].

#### Covariates

Seven variables (age, level of education, marital status, current working status, place of residence, wealth quintile and media exposure) were considered in this study as covariates. We utilised the existing coding for age, educational level, current working status, place of residence, and wealth quintile as found in the DHS dataset. In the DHS, age was coded as ‘15–19’, ‘20–24’, ‘25–29’, ‘30–34’, ‘35–39’, ‘40–44’, and ‘45–49’. The level of education was coded as ‘no education’, ‘primary’, ‘secondary’, and ‘higher’. Using Principal Component Analysis (PCA), the wealth quintile in the DHS was analysed as an index of household assets and utilities and classified as “poorest,” “poorer,” “middle,” “richer,” and “richest.” The original wealth quintile categorization employed in the DHS was used in this study. Place of residence was coded as ‘urban’ and ‘rural’. Current working status was coded as ‘no’ and ‘yes’. Media exposure was generated from three variables on the frequency of watching television, reading newspaper/magazine, and listening to radio. Each of these variables were categorized into “not at all, less than once a week, at least once a week, and almost every day”, which were re-categorized into ‘No’ (not at all) and “Yes” (less than once a week, at least once a week, and almost every day). An index variable called mass media exposure was created using the recoded responses from the three variables. Any woman whose response option was “Yes” in any of the variables after the recoding was said to have been exposed to mass media whilst those that responded “No” in all the three were said to have no exposure to mass media.

### Data analyses

Different data analyses were carried out using Stata version 16.0. For the first analysis, the prevalence of IPV and the proportion of women who had sexual autonomy were calculated from frequencies and percentages and were presented by bar charts. Second, Pearson’s chi-square test of independence was used to examine the independent associations between sexual autonomy and IPV (involving physical violence, emotional violence, and sexual violence) in each of the 24 countries included in this study. Finally, the association between sexual autonomy on IPV in each of the 24 countries was assessed through bivariable and multivariable binary logistic regression models. Two models (Model I and II) were built to examine the association between sexual autonomy and IPV. The first model (bivariable) consisted of only the sexual autonomy and IPV. In the second model (multivariable), we included all the covariates together with sexual autonomy and IPV. The results were presented as crude odds ratios (ORs) and adjusted odds ratios (AORs), at 95% confidence intervals (CIs). The women’s sample weights for the domestic violence module (d005/1,000,000) were applied to get unbiased estimates, according to the DHS guidelines. Also, the survey command (svy) in Stata was used to adjust for the complex sampling structure of the data in the regression analyses.

### Ethical consideration

Ethical approval was not sought for thestudy since the analysis was done using publicly available data. Details about data and ethical standards are available at: http://goo.gl/ny8T6X.

## Results

### Background characteristics of the women in sub-Saharan Africa

Out of the 99,769 women, majority (21.4%) were aged 25–29 years. Most of the women had no education (37.1%), were married (78.2%), and exposed to mass media (66.8%). More than half (66.8%) of the women were currently working. Most (20.3%) of the women belonged to the poorer wealth quintile. Majority (64.2%) of the women were residing in rural areas (see Table 2).

**Table 2:** Distribution of IPV across sexual autonomy and covariates among women in SSA

Variable	Weighted N	Weighted %	IPV		
			No (%)	Yes (%)	p-value
Sexual autonomy					< 0.001
No	26,933	27.0	66.9	33.1	
Yes	72,836	73.0	59.5	40.5	
Maternal age					< 0.001
15–19	6262	6.3	70.5	29.5	
20–24	17,445	17.5	61.9	38.1	
25–29	21,402	21.4	60.2	39.8	
30–34	19,243	19.3	60.4	39.6	
35–39	15,723	15.8	59.7	40.3	
40–44	11,360	11.4	62.4	37.6	
45–49	8335	8.3	61.4	38.6	
Maternal educational level					< 0.001
No education	37,040	37.1	66.0	34.0	
Primary	32,600	32.7	55.7	44.3	
Secondary	25,456	25.5	62.2	37.8	
Higher	4673	4.7			
Marital status					< 0.001
Married	77,973	78.2	63.1	36.9	
Cohabiting	21,796	21.8	55.6	44.4	
Current working status					< 0.001
No	33,187	33.3	65.9	34.1	
Yes	66,582	66.7	59.3	40.7	
Exposed to mass media					0.111
No	33,093	33.2	60.9	39.1	
Yes	66,676	66.8	61.8	38.2	
Wealth index					< 0.001
Poorest	19,051	19.1	59.2	40.8	
Poorer	20,314	20.3	59.1	40.9	
Middle	20,145	20.2	60.8	39.2	
Richer	20,224	20.3	61.6	38.4	
Richest	20,036	20.1	66.7	33.3	
Place of residence					0.051
Urban	35,731	35.8	62.3	37.7	
Rural	64,038	64.2	61.0	39.0	

*SD* standard deviation

### Prevalence of IPV and sexual autonomy among women in sub-Saharan Africa

**Fig. 1:**
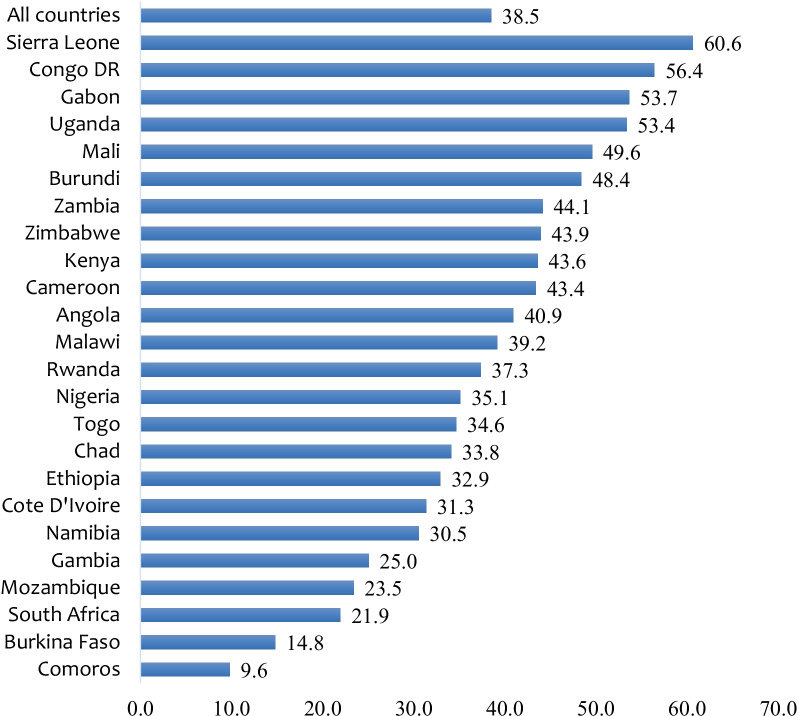
Prevalence (%) of IPV among married and cohabiting women in SSA

**Fig. 2:**
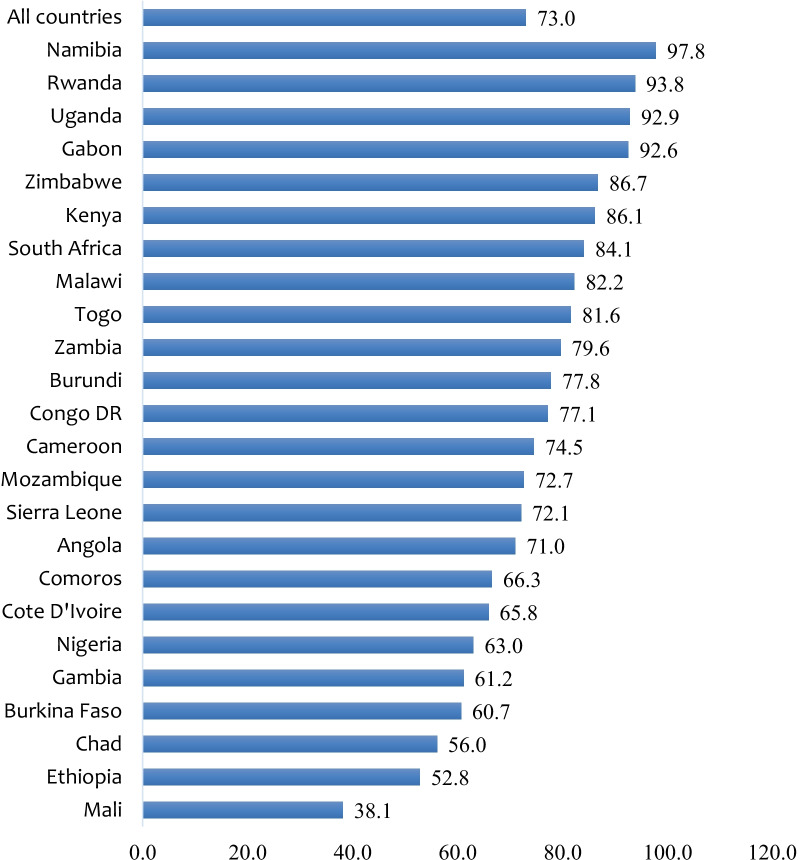
Prevalence (%) of sexual autonomy among married and cohabiting women in SSA

In the 24 sub-Saharan African countries studied, the prevalence of IPV was 38.5%. On the country level, the highest prevalence of IPV was 60.6% in Sierra Leone with the lowest in Comoros (9.8%) (Fig. 1). In terms of sexual autonomy among married and cohabiting women, the overall prevalence was 73.0%. Married and cohabiting women in Namibia had the highest prevalence of sexual autonomy with 97.8% and the lowest recorded in Mali (38.1%) (see Fig. 2).

### Distribution of sexual autonomy among married and cohabiting women and IPV in Sub-Saharan Africa

Table 3 shows the distribution of sexual autonomy among married and cohabiting women across PV, EV, SV and IPV by countries in Central, West, East and Southern Africa. Sexual autonomy showed a significant association with PV, EV, SV, and overall IPV in SSA. In terms of specific violence, Sierra Leone recorded the highest PV (50.8%) and EV (44.1%) among those with sexual autonomy. Congo DR recorded the highest SV (24.0%) among women with sexual autonomy. Sexual autonomy had significant association with IPV in 13 countries (Angola, Cameroon, Chad, Gabon, Cote d’lvoire, Gambia, Nigeria, Burundi, Kenya, Uganda, Namibia, South Africa, and Zambia) out of the 24. Thus, in these countries, IPV was higher among married and cohabiting women who had sexual autonomy.

**Table 3:** Sexual autonomy and physical, emotional, sexual, and intimate partner violence by countries

Countries	Ever experienced PV		p-values	Ever experienced EV		p-values	Ever experienced SV		p-values	Ever experienced IPV		p-values
	No	Yes		No	Yes		No	Yes		No	Yes	
All countries	23.9	29.2	< 0.001	22.6	27.1	< 0.001	8.3	11.4	< 0.001	33.1	40.5	< 0.001
Central Africa												
Angola	24.0	35.2	< 0.001	17.8	30.6	< 0.001	5.7	8.5	0.003	30.1	45.3	< 0.001
Cameroon	27.5	35.5	0.001	23.5	28.6	0.024	6.3	10.4	0.022	35.9	46.0	< 0.001
Chad	19.0	30.5	< 0.001	19.1	27.2	< 0.001	6.4	11.5	< 0.001	27.3	38.9	< 0.001
Congo DR	44.3	44.4	0.791	35.1	34.9	0.867	24.5	24.0	0.517	54.2	57.0	0.485
Gabon	36.7	45.1	0.183	23.2	33.6	0.115	10.9	14.4	0.365	40.3	54.8	0.024
West Africa												
Burkina Faso	9.8	11.0	0.187	7.9	9.4	0.090	1.1	1.4	0.431	13.8	15.4	0.128
Cote d’lvoire	18.7	28.7	< 0.001	15.8	20.7	0.010	3.1	6.7	0.001	24.7	35.0	< 0.001
Gambia	15.1	20.7	0.015	11.2	17.0	0.004	1.7	2.7	0.201	19.8	28.3	0.001
Mali	36.6	38.3	0.466	36.9	42.0	0.049	9.6	16.1	< 0.001	47.8	52.5	0.068
Nigeria	15.2	19.3	0.001	29.4	31.3	0.191	6.7	6.6	0.923	32.9	36.4	0.023
Sierra Leone	46.3	50.8	0.131	46.9	44.1	0.357	8.1	7.9	0.900	57.8	61.7	0.148
Togo	21.4	18.8	0.195	32.5	28.1	0.068	9.7	6.5	0.012	37.6	34.1	0.184
East Africa												
Burundi	40.6	36.9	0.034	25.1	21.5	0.018	27.7	23.1	0.003	52.1	47.3	0.012
Comoros	3.5	5.2	0.178	6.6	7.8	0.560	1.0	1.8	0.350	7.5	10.7	0.150
Ethiopia	24.5	20.4	0.072	23.2	23.2	0.976	10.0	9.4	0.710	34.3	31.7	0.352
Kenya	29.8	34.6	0.094	21.9	29.9	0.004	7.2	12.5	0.006	35.1	45.0	0.001
Mozambique	18.8	17.3	0.609	13.2	15.1	0.408	3.0	3.3	0.829	23.9	23.3	0.866
Rwanda	30.6	28.4	0.621	21.6	22.2	0.956	18.7	9.2	0.002	40.4	37.1	0.294
Uganda	45.7	36.6	0.001	43.4	37.7	0.057	24.4	21.1	0.187	62.0	52.7	0.002
Southern Africa												
Malawi	21.8	24.0	0.290	21.8	26.9	0.018	16.3	18.0	0.364	36.7	39.7	0.207
Namibia	50.3	20.7	0.003	38.2	22.2	0.106	35.5	5.9	< 0.001	58.5	29.9	0.016
South Africa	9.9	14.0	0.136	10.3	16.3	0.022	3.0	3.7	0.708	14.8	23.3	0.010
Zambia	33.4	34.4	0.645	24.0	28.8	0.006	10.9	14.1	0.009	40.7	45.0	0.028
Zimbabwe	29.3	29.3	0.980	27.7	30.5	0.241	8.5	12.0	0.078	40.0	44.5	0.090

Pearson chi-square test was used to obtain p-values

*PV* physical violence, *EV* emotional violence, *SV* sexual violence, *IPV* intimate partner violence

### Association between sexual autonomy among married and cohabiting women and IPV in sub-Saharan Africa

The logistic regression analysis also showed significant association between sexual autonomy and IPV among women in sub-Saharan Africa. This, however, does not support our hypothesis that women’s sexual autonomy is inversely associated with risk of experiencing IPV. The odds of having IPV were higher among women with sexual autonomy [OR = 1.38, 95% CI = 1.31–1.44] but this reduced slightly after controlling for the confounders [aOR = 1.28, 95% CI = 1.21–1.35]. In the adjusted model, sexual autonomy had significant association with IPV among women in Angola, Cameroon, Chad, and Gabon in Central Africa. In West Africa, the odds of IPV were higher among women with sexual autonomy in Cote d’lvoire, Gambia, Mali, and Nigeria. In East Africa, sexual autonomy had significant association with IPV among women in Burundi, Comoros, and Kenya. Finally, in Southern Africa, sexual autonomy had significant association with IPV among women in South Africa, and Zambia (see Model II of Table 4).

**Table 4:** Logistic regression analysis on the association between sexual autonomy and IPV among women in sub-Saharan Africa

Countries	Model I	Model II
	OR [95%CI]	aOR [95%CI]
All countries	1.38*** [1.31–1.44]	1.28*** [1.22–1.35]
Central Africa		
Angola	1.64*** [1.46–1.83]	1.65*** [1.45–1.86]
Cameroon	1.51*** [1.29–1.76]	1.24* [1.05–1.46]
Chad	2.09*** [1.73–2.51]	1.89*** [1.55–2.30]
Congo DR	1.10 [0.96–1.25]	1.09 [0.95–1.23]
Gabon	1.97*** [1.54–2.53]	1.84*** [1.42–2.39]
West Africa		
Burkina Faso	1.14* [1.01–1.28]	1.09 [0.97–1.23]
Cote d’lvoire	1.66*** [1.44–1.91]	1.61*** [1.39–1.87]
Gambia	1.54*** [1.31–1.81]	1.58*** [1.33–1.87]
Mali	1.26** [1.09–1.46]	1.19* [1.02–1.39]
Nigeria	1.14** [1.04–1.26]	1.17** [1.05–1.31]
Sierra Leone	1.14 [0.99–1.32]	1.03 [0.88–1.19]
Togo	0.84* [0.73–0.98]	0.95 [0.81–1.11]
East Africa		
Burundi	0.78*** [0.69–0.88]	0.83** [0.73–0.94]
Comoros	1.54* [1.11–2.16]	1.49* [1.06–2.10]
Ethiopia	0.98 [0.85–1.12]	1.06 [0.92–1.23]
Kenya	1.63*** [1.37–1.96]	1.57*** [1.29–1.91]
Mozambique	1.15 [0.88–1.49]	0.83 [0.63–1.11]
Rwanda	0.80 [0.52–1.21]	0.82 [0.53–1.27]
Uganda	0.74** [0.61–0.89]	0.86 [0.70–1.04]
Southern Africa		
Malawi	1.13 [0.97–1.33]	1.15 [0.98–1.35]
Namibia	0.43* [0.21–0.87]	0.55 [0.26–1.14]
South Africa	1.94*** [1.38–2.72]	1.88*** [1.32–2.66]
Zambia	1.20** [1.06–1.36]	1.28*** [1.13–1.45]
Zimbabwe	1.15 [0.97–1.36]	1.17 [0.99–1.39]

Model1: unadjusted model examining the independent association between sexual autonomy and IPV; Model II: adjusted for age, wealth, educational level, place of residence, marital status, current working status, and media exposure); OR is the odds ratio, aOR is the adjusted odds ratio. Reference categories were no intimate partner violence

*p < 0.05

**p < 0.01

***p < 0.001

## Discussion

This study sought to determine the association between sexual autonomy and IPV among women in sexual unions in SSA. We found the pooled prevalence of sexual autonomy and IPV to be 73.0% and 38.5% respectively. Women who had sexual autonomy were more likely to experience IPV using the pooled data.

The pooled prevalence of IPV among the women in our study is comparable with the findings of previous studies [26, 30]. At the country level, the highest prevalence of IPV was 60.6% in Sierra Leone. This finding is not surprising since conflict and post-conflict situations increase the susceptibility of women to violence due to the deterioration of social protection measures during such times [8, 31, 32]. Another possible reason for the high prevalence of IPV in Sierra Leone could be  explained by some socio-cultural factors. For instance, Horn et al. [33] revealed that culture and religion in Sierra Leone encourages women to remain in violent relationships. The idea is that women must remain in such relationships, to avoid leaving the children in the care of “wicked” stepmothers. Additionally, the use of IPV as a proxy punishment for women’s refusal to perform their conjugal duty, which is only excusable in a case of sickness could have accounted for the high prevalence of IPV [34]. Our study also recorded the lowest prevalence of IPV in Comoros, and this agrees with the finding of Izugbara et al. [8] that women in Comoros were the safest when it comes to IPV in SSA.

For sexual autonomy among married and cohabiting women, the overall prevalence was 73.0%. While this figure is relatively high, there exists cross-country differences that suggest that many countries in the sub-region still have lower rates of sexual autonomy among women. For example, while married and cohabiting women in Namibia recorded as high as 97.8% sexual autonomy, those in Mali recorded 38.1%. In effect, it is important for policies aimed at improving women’s sexual autonomy in the sub-region to pay critical attention to cross-country variations. Countries with low sexual autonomy among women deserve greater attention.

In this study, we found a significant association between sexual autonomy and IPV among women in SSA. This, however, did not support the hypothesis that women’s sexual autonomy is inversely associated with risk of experiencing IPV. Interestingly, the odds of IPV among women in sexual unions were higher among those with sexual autonomy compared to those without sexual autonomy. This finding is consistent with report from other study [26]. A study by Sunmola et al. [20] found that women with the ability to negotiate safer sex with their partners were more likely to experience more forms of violence in Nigeria. This could be that women with sexual autonomy would have the capacity to fight for their rights, which their husbands will interpret as a challenge to their authority, making them act violently towards their wives. Also, women with sexual autonomy would tend go against the cultural norms that preach subordination of women in intimate relationships, which may result in IPV. In most African settings, women cannot refuse or deny their partners sex unless they are menstruating, pregnant, breastfeeding, or are in their betrothal period. Hence, any attempt by a woman to deny or refuse sex is likely to be subjected to violence [35]. Deep-rooted cultural norms could have accounted for significant finding in our study. For example, cultural norms demand that women should conform and submit to their husbands’ sexual desires and demands without hesitation could have perpetrated IPV in situations where women refuse or try to negotiate for a safer sex [20].

Another important aspect of IPV that needs to be noted in terms of sexual violence and sexual autonomy is that women, who do not feel they have the right or are empowered to ask their partners to use condoms or refuse sex, have the tendency not to disclose sexual violence to people for legal action. This is due to the fact that in many African communities, sexual violence remains a sensitive topic to be discussed which is seen as a taboo for women to talk about it with their husband in some cultures. This, however, makes victims of sexual violence feel uncomfortable to report it in studies [36]. Therefore, studies on association between sexual autonomy and IPV need to be interpreted with caution.

## Strengths and limitation

The study has some strengths and limitations. The main strength of the study lies in its use of nationally representative datasets of many countries in SSA. This notwithstanding, the study adopted a cross-sectional design, which prevents us from making causal inferences between the studied variables. Also, given the retrospective nature of reporting, which characterizes DHS data, this study is not immune to recall biases. Also, given the socio-cultural norms that surround issues of IPV in some countries, the data may be subject to social desirability biases. Furthermore, because the data was collected at different times, there could have been changes within and across countries that muddled the findings of this study. Hence, the generalization of the findings to all women in the respective countries should be done with caution.

## Conclusion

This study found a significant association between the association between sexual autonomy and IPV among women in sub-Saharan Africa. This, however, did not support our hypothesis that women’s sexual autonomy is inversely associated with risk of experiencing IPV. The overall prevalence of IPV among women in 24 sub-Saharan African countries was 38.5% and that of sexual autonomy among married and cohabiting women was 73.0%. Moreover, sexual autonomy had significant association with IPV in 13 countries (Angola, Cameroon, Chad, Gabon, Cote d’lvoire, Gambia, Nigeria, Burundi, Kenya, Comoros, Uganda, South Africa, and Zambia). We suggest that social interventions aimed at empowering women in SSA must pay particular attention to countries with high rates of IPV. Based on our findings, there is the need for sub-Saharan African countries to step up programs that will improve IPV reporting and access to legal support for those who experience IPV. Despite the relevance of the study’s findings, we recommend that caution must be taken when interpreting the results on the association between the sexual autonomy and IPV since there is reporting bias concerning sexual violence.

## Supplementary Information

**Additional file 1: Table S1.** STROBE 2007 (v4) Statement—Checklist of items that should be included in reports of cross-sectional studies.

## Data Availability

The dataset is freely accessible via this link: https://dhsprogram.com/data/available-datasets.cfm.

## Abbreviations

AOR: Adjusted odds ratio
CI: Confidence interval
COR: Crude odds ratio
DHS: Demographic and Health Survey
IPV: Intimate partner violence
SDGs: Sustainable Development Goals
SSA: Sub-Saharan Africa
